# Overcoming challenges to screening and brief intervention implementation with technology: the promise of interactive voice response systems

**DOI:** 10.1186/1940-0640-7-S1-A93

**Published:** 2012-10-09

**Authors:** Gail Rose, John Helzer

**Affiliations:** 1 Health Behavior Research Center, University of Vermont, Burlington, VT, USA; 2 Department of Psychiatry, Health Behavior Research Center, University of Vermont, Burlington, VT, USA

## 

While the efficacy of screening and brief intervention (SBI) is supported by multiple clinical trials, routine delivery of SBI is hampered by complex factors, both individual and systemic. An alternative to the traditional in-office approach to SBI holds promise as a way of broadening its reach. The purpose of this study is to share our experience using interactive voice response (IVR) technology to deliver and augment SBI in primary care. We draw on experience from a 12-year program of research in this area, summarizing results from prior studies and describing a new trial currently underway. Questions regarding the nature of IVR; how it works; why it might be a helpful modality for fostering, delivering, and/or augmenting SBI; and how others can embark on IVR research are addressed. We provide a brief overview of the IVR applications we have employed in alcohol research, a description of some methodological challenges we encountered in a prior SBI trial, and how these experiences led us to develop our current IVR-based SBI program. We cover technicalities of developing IVR applications and issues of their implementation in research. Interactive voice response is a feasible modality for delivering assessment and advice about drinking. The technology required for implementation is accessible to researchers and clinicians who do not necessarily have specialized training or expertise. Although this technology offers several advantages, including patient comfort with disclosure of personal information and widespread access and availability, it has downsides as well. For example, refusal and noncompliance can be higher than in other modalities. A small body of research suggests that process variables such as interviewer voice characteristics, survey length, and questionnaire-item format may influence consent to participate and compliance with IVR surveys and interventions.

